# Expression of Gata Binding Protein 3 as a Prognostic Factor in Urogenital Lesions and Its Association With Morphology

**DOI:** 10.7759/cureus.49635

**Published:** 2023-11-29

**Authors:** T Govardhan, Debahuti Mohapatra, Sujata Naik, Prateek Das, Pranita Mohanty, Ankita Pal

**Affiliations:** 1 Pathology, Apollo Institute of Medical Sciences and Research, Chittoor, IND; 2 Pathology, Institute of Medical Sciences and SUM Hospital, Bhubaneswar, IND; 3 Haematooncopathology, Tata Memorial Centre Varanasi, Homi Bhabha Cancer Hospital and Mahamana Pandit Madan Mohan Malaviya Cancer Centre, Varanasi, IND; 4 Pathology, Chirayu Medical College and Hospital, Bhopal, IND

**Keywords:** gata3, renal cell carcinoma, prostate cancer, urothelial carcinoma, histopathology, immunohistochemistry

## Abstract

Background: Urogenital malignancies, encompassing urinary bladder cancer, prostate cancer, and renal cell carcinoma, pose significant diagnostic challenges due to overlapping histopathological features. GATA binding protein 3 (GATA3), a transcription factor associated with urothelial tissue, has shown promise as a potential diagnostic marker. This study aimed to investigate the incidence of these malignancies, explore GATA3's involvement in urothelial cancer (UC), and determine its role in distinguishing urogenital malignancies.

Materials and methods: A cross-sectional, retro-prospective, hospital-based study was conducted from May 2019 to April 2021. The surgical samples of patients who underwent transurethral resection of bladder tumour (TURBT), transurethral resection of the prostate (TURP), radical cystoprostatectomy, total and partial radical nephrectomy specimens during the study period were reviewed. Patients diagnosed with urinary bladder neoplasm and high-grade prostate neoplasm along with chromophobe, oncocytic, sarcomatoid variant and clear cell carcinoma, renal cell carcinoma were included. Immunohistochemical analysis of GATA3 expression was performed, with scoring based on nuclear staining intensity and percentage of tumor cells labeled.

Results: The study included 64 patients, predominantly males over 60 years. Personal habits revealed a high prevalence of smoking (85.9%). The most prevalent symptom was hematuria (75.0%), followed by hematuria with urgency (20.3%). The most common site of lesion was posterolateral (31.3%). Urothelial cancer was the most common malignancy, primarily high-grade. Strong positive GATA3 expression was significantly associated with high-grade UC (p=0.01) and invasion (p=0.01). However, low-grade UC and papillary urothelial neoplasm of low malignant potential exhibited moderate GATA3 expression. GATA3 demonstrated potential for distinguishing UC from other histological types.

Conclusion: GATA3 expression correlates with high-grade urothelial cancer and invasive behavior, suggesting its utility as a diagnostic marker in challenging cases.

## Introduction

Urinary bladder cancer is a prevalent malignancy that affects the urinary system, with nearly 98% of malignant bladder tumors originating from epithelial tissue [1]. Among these tumors, around 90% are categorized as urothelial carcinomas (UC) [2]. Numerous demographic studies have demonstrated that individuals aged 65 years or older experience an incidence rate that is 11-fold higher compared to those below 65 years of age [3]. Also, it is three times more common in men than in women [2]. The increased incidence is due to risk factors such as smoking and occupational exposure to aromatic amines [4].

Differentiating between poorly differentiated prostate cancer and high-grade UC in transurethral resection samples presents a commonly encountered challenge [5]. This complexity is heightened by the frequent occurrence of glandular differentiation in the latter and the often-elevated levels of prostate-specific antigen when UC extends into the prostate gland. When it comes to distinguishing prostate carcinoma from bladder cancer, particularly in cases where the tumor is localized to the bladder neck or extends into the surrounding prostatic tissue, the task becomes even more intricate. This distinction holds substantial therapeutic [5] and staging implications since accurately determining tumor grade and the stage is imperative for prognostication, necessitating precise diagnoses to ascertain the extent of both bladder and prostate cancer.

Despite examining various indicators to identify whether poorly differentiated tumors originate from the prostate or urothelial tissues, no marker has proven adequately sensitive or specific thus far. While prostate-specific antigen and prostate-specific acid phosphatase have conventionally been employed to validate a prostate origin, there are instances when they might show limited expression, focal presence, or even absence in poorly differentiated primary and metastatic prostate adenocarcinomas [6]. GATA binding protein 3 (GATA3) has proven to play an important role in promoting and directing cell proliferation, development, and differentiation in many tissues and cell types [7]. GATA3, associated with urothelial tissue, has demonstrated utility in aiding the distinction between different types of UC [7,8]. This study aims to investigate the incidence of different urogenital malignancies and explore GATA3's involvement in UC.

## Materials and methods

Study design and setting

This cross-sectional, hospital-based, retro-prospective study was conducted in the Department of Pathology, Institute of Medical Sciences and SUM Hospital, Bhubaneswar. It was carried out for a duration of three years (one-year retrospective, May 2018 to April 2019, and two years prospective, May 2019 to April 2021). This study was conducted in accordance with ethical principles that are consistent with the Declaration of Helsinki. The study protocol was approved by the Institutional Review Board/Ethics Committee (REF. NO./DMR/IMSSH/SOA/180369). Written informed consent was obtained from all the patients prior to study commencement.

Inclusion/exclusion criteria

The surgical samples of patients who underwent transurethral resection of bladder tumour (TURBT), transurethral resection of the prostate (TURP), radical cystoprostatectomy, total and partial radical nephrectomy specimens in the department of urology from May 2018 to April 2021 were reviewed. Patients diagnosed with urinary bladder neoplasm and high-grade prostate neoplasm along with chromophobe, oncocytic, sarcomatoid variant, and clear cell carcinoma, renal cell carcinoma were included. Samples from both retrospective and prospective studies were collected, along with clinical data, and paraffin-embedded blocks were retrieved from the records in the Department of Pathology. Patients undergoing cystoscopic biopsy for urinary bladder neoplasm were also included. The study excluded patients with poorly differentiated renal cell carcinoma and testicular tumors. Additionally, patients with acute painful conditions of the urinary bladder, those lacking supportive clinical and radiological reports, patients with issued paraffin blocks for further evaluation, instances where patient consent was not obtained, and those who could not be followed due to the coronavirus disease 2019 (COVID-19) situation were also excluded.

Evaluation of immunohistochemical (IHC) slides (path in situ)

The immunohistochemical marker GATA3, using the L50-823 monoclonal antibody as a positive control, demonstrates nuclear positivity in bladder transitional cell carcinoma, aiding in the identification of this specific cancer type.

Immunohistochemical (IHC) scoring [8]

Nuclear staining for GATA3 is considered positive; the percentage of tumor cells labelled by GATA3 was scored as follows. Score 0: No tumor cells stained; Score 1: 1-10%; Score 2: 11-50%; Score 3: 51-80%; Score 4: 81-100%. The staining intensity of tumor cells labelled by GATA3 was scored as follows. Staining score 0: No tumor cells stained; Staining score 1: Weak; Staining score 2: Moderate; Staining score 3: Strong.

Grouping of GATA3 along with interpretation [8,9] is given as follows. Group I (Score 0-1): Negative; Group II (Score 2-4): Weakly positive; Group III (Score 5-8): Moderately positive; Group IV (Score 9-12): Strongly positive.

Statistical analysis

All the statistical analysis of both histopathology and IHC features was done by using SPSS software, version 22.0 (IBM Corp., Armonk, NY). Data was checked for completeness and consistency. Descriptive statistics summarize categorical variables using frequency and percentages. Categorical variables were analyzed using Chi square test. A p-value less than 0.05 was considered statistically significant.

## Results

The study involved a total of 64 patients, with 46.8% having prostate cancer, 39.1% diagnosed with urinary bladder cancer, and 14.1% experiencing renal cell carcinoma. Table 1 summarizes the baseline characteristics of the study population. The most common site of lesion was the posterolateral urinary bladder (31.3%), followed by the lateral (26.6%) and the least common was the anterior wall (20.3%) and others (21.9%) (Table 1).

**Table 1:** Baseline characteristics of the study population Data presented as n (%). Others: apex (2), base (1), metastatic (2), neck (2), posterior (6), vesicoureteric junction (VUJ) (1).

Parameters	Number of patients (N=64)
Age (years)	
21-40	1 (1.5)
41-60	23 (35.9)
>60	40 (62.5)
Gender	
Male	56 (87.5)
Female	8 (12.5)
Personal habits	
Smoking	55 (85.9)
Alcohol	9 (14.1)
Signs & Symptoms	
Hematuria	48 (75.0)
Hematuria +Urgency	13 (20.3)
Urgency	3 (4.7)
Site of lesions	
Posterolateral	20 (31.3)
Lateral	17 (26.6)
Anterior	13 (20.3)
Others	14 (21.9)

Urothelial cancer was observed in 49 patients (76.5%). In this study, high-grade UC was seen in 75.5% patients and low-grade UC in 18.4% patients. In patients with high-grade UC, significantly higher number of patients (72.7%) showed strong positive GATA3 expression than those with low-grade UC (27.3%, p=0.01). In patients with papillary urothelial neoplasm of low malignant potential (PUNLMP), all the patients (n=3) showed a moderate positive expression of GATA3 (Table 2). 

**Table 2:** GATA3 expression with grade, invasion, and necrosis in patients with UC Data presented as n (%). LVI, lymphovascular invasion; PNI, perineural invasion; PUNLMP, papillary urothelial neoplasm of low malignant potential, UC, urothelial cancer.

Parameters	Total (n=49)	Negative (n=15)	Weak positive (n=5)	Moderate positive (n=7)	Strong positive (n=22)
Grade					
High	37 (75.5)	12 (80.0)	5 (100)	4 (57.1)	16 (72.7)
Low	9 (18.4)	3 (20.0)	0	0	6 (27.3)
PUNLMP	3 (6.1)	0	0	3 (42.8)	0
Invasion					
Lamina	11 (22.4)	1 (6.7)	0	2 (28.5)	8 (36.4)
Muscle	31 (63.3)	13 (86.7)	3 (60.0)	2 (28.5)	13 (59.1)
Non-invasive	7 (14.3)	1 (6.7)	2 (40.0)	3 (42.8)	1 (4.5)
Necrosis	12 (24.5)	5 (33.3)	1 (20.0)	1 (14.3)	5 (22.7)
LVI	31 (63.3)	13 (86.7)	3 (60.0)	2 (28.6)	13 (59.1)
PNI	6 (12.2)	2 (13.3)	0	1 (14.3)	3 (13.6)

**Figure 1:**
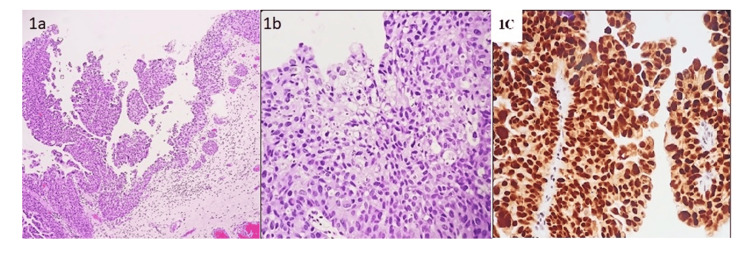
Morphological characteristics and GATA3 expression in the tumour with papillary and congested blood vessels in the lamina propria (1a) Photomicrograph shows a tumour with papillary structures and congested blood vessels in the lamina propria (H&E,10x); (1b) The image shows transitional cells exhibiting moderate to marked pleomorphism with overcrowding and loss of polarity. The cells have round to oval enlarged conspicuous hyperchromatic nuclei, conspicuous nucleoli, and moderate amounts of cytoplasm. Increased mitotic activity is seen at different levels of stratification (H&E,40x); (1c) shows strong nuclear positivity.

**Figure 2:**
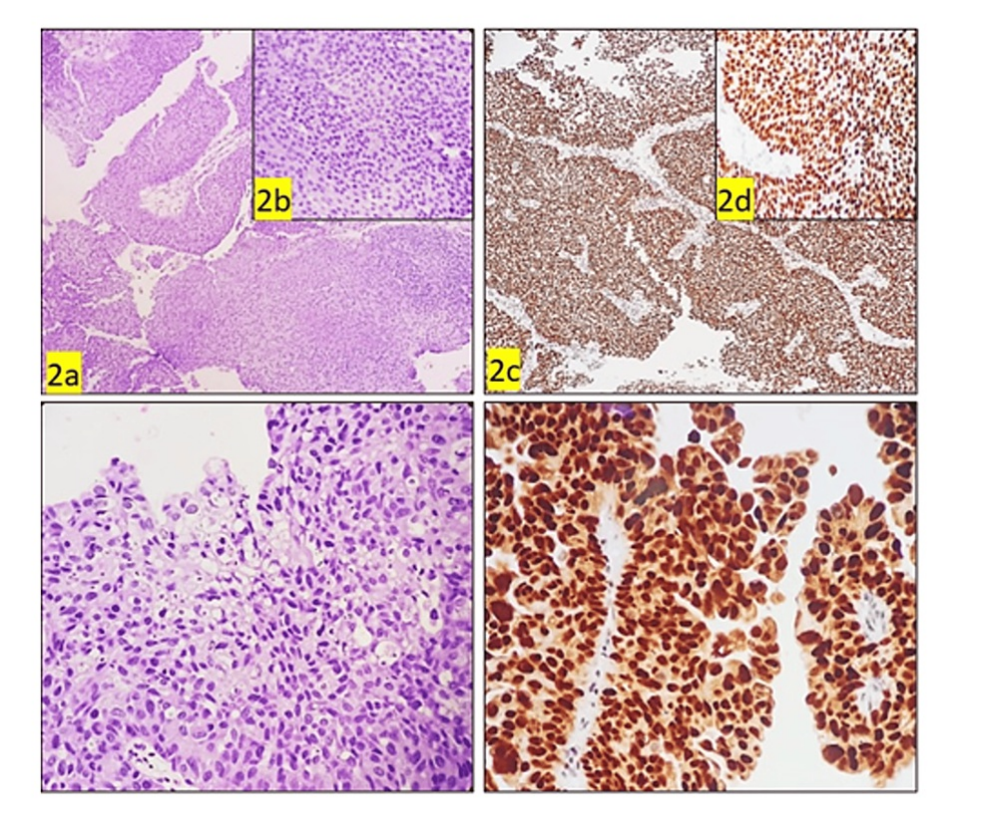
Morphological characteristics and GATA3 expression in LGUC LGUC, low-grade urothelial cancer; GATA3, GATA binding protein 3 (2a, 2b) The image shows an increased cell layer, predominantly ordered architectural pattern of fused papillae showing moderate pleomorphism, (LGUC) (H&E, 10x & 40x); (2c, 2d) (GATA3) showing strongly positive (H&E, 10x & 40x); (2e) the image shows cells exhibiting moderate to marked pleomorphism with overcrowding and loss of polarity. The cells have round to oval enlarged conspicuous hyperchromatic nuclei, conspicuous nucleoli, and moderate amounts of cytoplasm (H&E,40x); (2f) (GATA3) showing strongly positive (H&E,40x).

**Figure 3:**
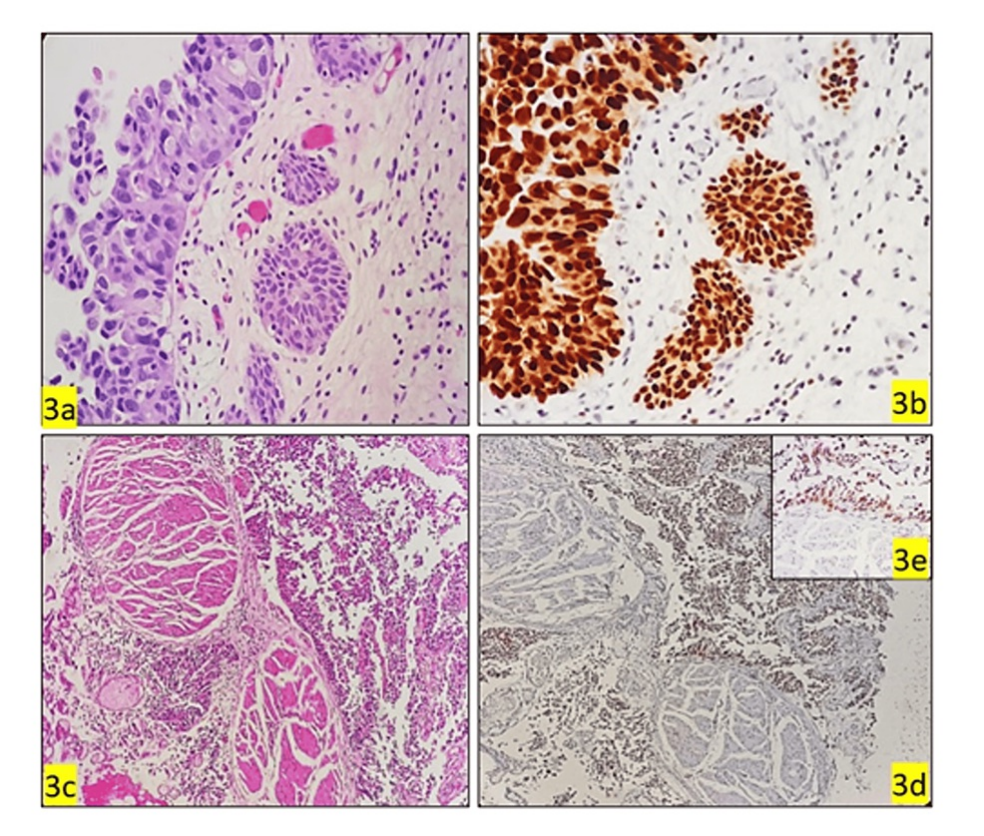
Morphological characteristics and GATA3 expression in HGUC invading lamina propria and muscularis propria GATA3, GATA binding protein 3; HGUC, high-grade urothelial cancer. (3a) Photomicrograph of HGUC shows neoplastic cells invading into lamina propria (H&E, 40x); (3b) (GATA3): Shows strongly positive (H&E, 40x); (3c) Photomicrograph of HGUC shows a neoplastic cell invading into muscularis propria (H&E, 10x); (3d, 3e) (GATA3) shows strongly positive (H&E, 10x & 40x).

**Figure 4:**
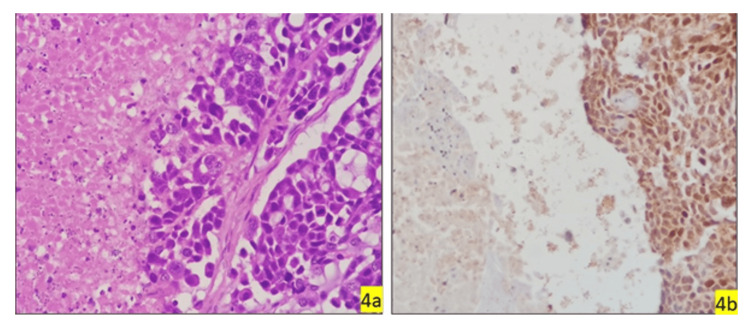
Necrosis and GATA3 expression in tumour microenvironment GATA3, GATA binding protein. (4a, 4b) (HPE & GATA3, 40x & 40x) shows necrosis and moderately positive.

**Figure 5:**
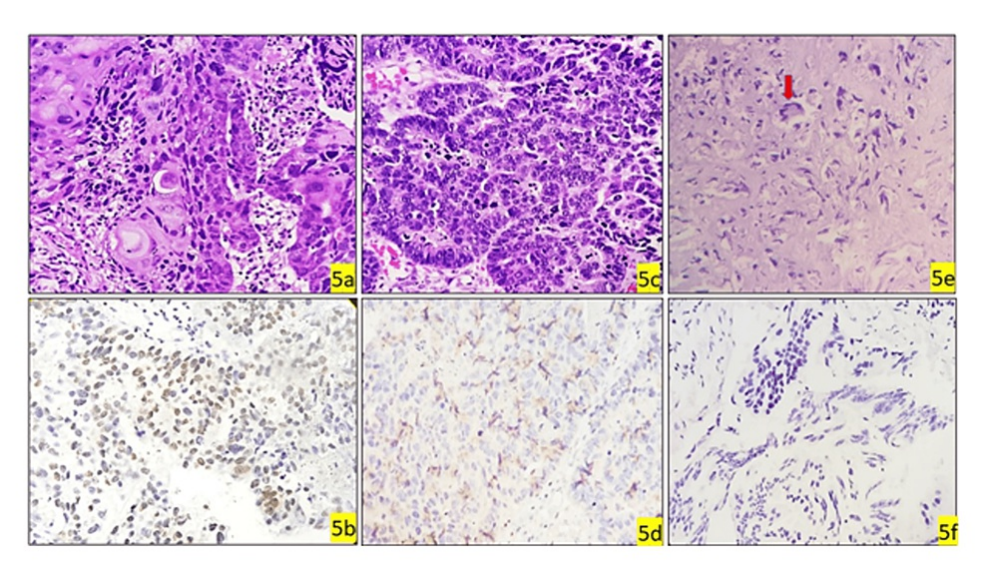
HGUC with squamous, neuroendocrine, and sarcomatous and subsequent GATA3 expression GATA-3, GATA binding protein 3; HGUC, high-grade urothelial cancer. (5a) HGUC with squamous differentiation; (5b) GATA3 shows moderately positive; (5c) HGUC with neuroendocrine differentiation; (5d) GATA3 shows negative; (5e) HGUC with sarcomatous differentiation; (5f) GATA3 shows negative.

**Figure 6:**
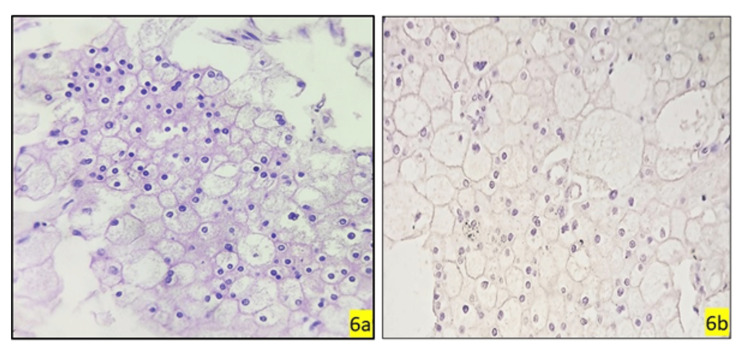
Morphological characteristics and GATA3 expression in chromophobe renal cell carcinoma GATA-3, GATA binding protein 3. (6a) Photomicrograph showing tumor cell arranged in sheets, comprised of polygonal to ovoid with well-delineated margins, small nuclei with conspicuous nucleoli, and abundant granular eosinophilic cytoplasm and infrequent mitotic figures (chromophobe renal cell carcinoma, H&E, 40x); (6b) (GATA3, 40x) Negative.

**Figure 7:**
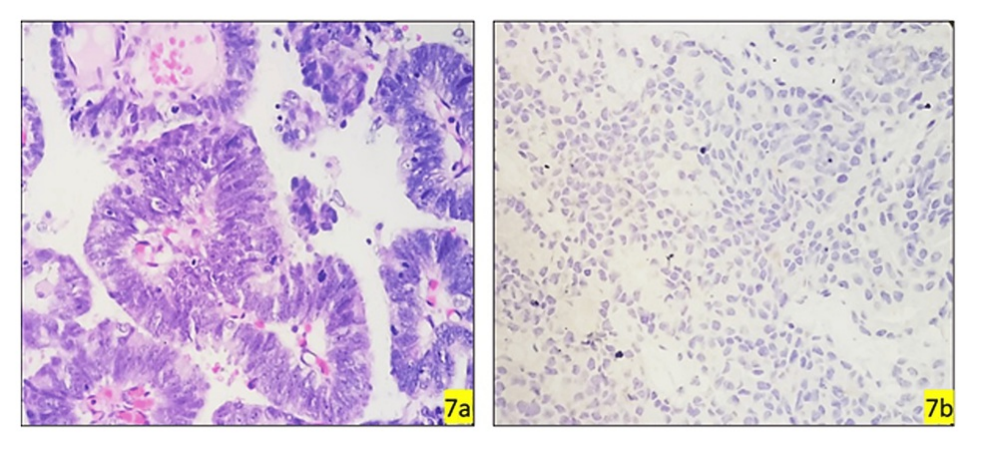
Ductal adenocarcinoma histopathology and GATA3 expression GATA3, GATA binding protein 3. (7a) Photomicrograph showing tumour cells arranged in a glandular and ductal pattern showing moderate pleomorphism, conspicuous nucleoli, moderate cytoplasm, and increased mitosis (ductal adenocarcinoma, H&E, 40x); (7b) (GATA3, 40x) Negative.

A significantly greater number of patients with invasion (muscle invasion, 59.1%, and laminar invasion, 36.4%) had a strong positive GATA3 expression than those with no invasion (4.5%, p=0.01). Necrosis was observed in 12 patients (24.5%), out of which strong positive expression of GATA3 was seen in most patients (n=5). Lymphovascular invasion (LVI) was present in 31 cases (63.3%) and an equal number of patients showed a negative and strong positive GATA3 expression (n=13 for both). Perineural invasion (PNI) was present in six patients (12.2%), out of these six patients, the majority showed a positive GATA3 expression (n=3) followed by negative GATA3 expression (n=2), and one patient with moderate GATA3 expression. Figures 1-7 illustrate the different morphological characteristics and GATA3 expression for different UC tumours.

## Discussion

The incidence of UC across the world is approximately 21.4%, involving urogenital sites like urinary bladder, kidney, cervix, uterus, ovary, prostate, and testis [10]. In India, the incidence of cancer at urogenital lesions is 20.7% [11]. Higgins et al. [12] were pioneers in studying the expression of GATA3 as an indicator for transitional epithelium and UC, and they proposed that it displayed a remarkably high level of specificity for UC. Furthermore, another study reported the specificity of GATA3 in prostate adenocarcinoma (PAC) was 15.2%, while in UC was 100% [5]. Therefore, this study aimed to explore the role of GATA3 in UC. 

In the present study, most patients with UC were in the age group >60 years, constituting 62.5% of patients. Similarly, in a study by Naik et al. [13], the average age of the participants was 61.28 years, with the majority (38.88%) falling within the age range of 61 to 70 years. Likewise, in another study, the average age at presentation was reported as 60.2 ± 4.4 years, and it was observed that low-grade cancer was more prevalent among patients below 60 years of age compared to those above 60 years of age (51.0% vs. 38.1%; p-value = 0.006) [2].

Bladder cancer is often more prevalent in men, and the differing occurrence and mortality rates between genders may be linked to differences in hormonal pathways [3]. In this study, a male predominance was observed (87.5%) which was similar to another study reporting that 80.8% of males were diagnosed with UC [13]. In these studies, the male-to-female ratios varied, with ratios of 4:1 [9] and 8.6:1 [2], respectively.

In the current study, smoking was observed in the majority of patients (87.5%) and alcohol consumption in 14.1% of patients. Another study has reported that the incidence of bladder cancer was four-fold higher in cigarette smokers [2]. Moreover, the study demonstrated that 74% of male participants and 22% of female participants with bladder cancer had a history of smoking or tobacco consumption in various forms [2].

In the current study, the majority of patients presented with hematuria (75.0%) and hematuria with urgency in 20.3% of patients. Gupta et al. [2] and Rana et al. [9] reported that 97.0% and 95.9% of the patients with bladder cancer presented with hematuria. In a study by Price et al. [14], macroscopic hematuria, the most common predictor for bladder cancers, exhibited a prevalence of 64% in patients aged ≥40 years diagnosed with bladder cancer.

In this study, the majority of patients had UC (76.5%), which was in parallel lines with another study conducted by Gupta et al. [2], having UC as the most common variant (97.7%). In the current study, a significantly higher proportion of patients with high-grade UC showed strong positive GATA3 expression than those with low-grade UC (p=0.01). Likewise, in another study, patients with low-grade UC (n=6), all of them exhibited strong positive GATA3 expression (100%) [13]. Additionally, among those with high-grade UC (n=116), 91 showed positive GATA3 expression, with 83 displaying strong to moderate expression and eight exhibiting weak expression [13]. On the contrary, GATA3 showed strong positive expression in all cases of low-grade UC (100%), and it was positive in 91 out of 116 cases (78.44%) of high-grade UC [13]. Rana et al. [9] revealed that all low-grade tumors displayed moderate to strong expression (100%), whereas most high-grade and invasive tumors exhibited weak or no expression (61.7%, p<0.001).

A significantly higher proportion of patients with tumor invasion showed strong positive than those without invasion in the present study (p=0.01). Additionally, muscular invasion was seen in 63.3% of patients, and laminar invasion in 22.4%. Likewise, a study conducted by Naik et al. [13] recorded invasive UC (n = 94), out of which 58.51% were muscle invasive and 41.48% were lamina propria invasive tumors. Similar to this study, Rana et al. [9] reported that GATA3 expression significantly correlated with muscle invasion (p=0.005).

In this study, necrosis was observed in 24.5% of patients with UC and an equal number of these patients showed negative and strong positive GATA3 expression (n=5 for both). In a study conducted by Chandra et al. [15], they documented a correlation between the presence of necrosis and GATA3 expression (p=0). Additionally, Agarwal et al. [8] found a significant statistical association between weak or absent GATA3 expression and the presence of necrosis (p = 0.019), which contrasted with the findings of this study.

Limitations

While this study provides valuable insights, it has limitations. The sample size may not fully represent the diversity of urogenital malignancies. Additionally, we encountered a limitation in instances where paraffin blocks were provided to patients for additional evaluation, but we were unable to pursue these cases due to the impact of the COVID-19 pandemic. Furthermore, the retrospective design introduces potential biases, and the exclusion of certain tumor types limits generalizability. Focusing solely on GATA-3 may not account for all relevant markers, and the study's duration may not capture long-term trends.

## Conclusions

In summary, this study highlights GATA3's potential in distinguishing urogenital malignancies, particularly high-grade urothelial carcinoma, from poorly differentiated prostate cancer. GATA3's strong positivity in high-grade urothelial carcinoma cases is promising for diagnostics. However, further research with larger and more diverse cohorts is needed to validate and expand upon these findings, potentially improving patient care in urogenital malignancies.
